# Concurrent multiple sclerosis and amyotrophic lateral sclerosis: where inflammation and neurodegeneration meet?

**DOI:** 10.1186/1742-2094-9-20

**Published:** 2012-01-24

**Authors:** Grace Li, Margaret M Esiri, Olaf Ansorge, Gabriele C DeLuca

**Affiliations:** 1 Clinical Medical School, University of Oxford, Oxford, UK; 2 Nuffield Department of Clinical Neurosciences (Neuropathology), University of Oxford, Oxford, UK

**Keywords:** multiple sclerosis, amyotrophic lateral sclerosis, neuropathology, inflammation, neurodegeneration

## Abstract

The concurrence of multiple sclerosis (MS) and amyotrophic lateral sclerosis (ALS) is exceedingly rare and the pathological features have not been examined extensively. Here we describe the key pathological features of a 40 year old man with pathologically confirmed concurrent MS and ALS.

This is the most pathologically illustrative case of coincident MS and ALS demonstrating inflammatory and neurodegenerative features characteristic of each disease, and is the first to exhibit the presence of TDP-43 inclusions in this clinical entity. The intricate relationship between neuroinflammation and neurodegeneration in these diseases is discussed.

## Background

MS has been traditionally viewed as an inflammatory demyelinating disease of the central nervous system with a secondary degenerative component whereas ALS has been considered a primary central and peripheral nervous system degenerative disorder with a secondary inflammatory component. In both diseases, neurological disability accrues secondary to axonal loss. While they appear on opposite ends of the inflammatory/degenerative spectrum, the relationship between neuroinflammation and neurodegeneration is likely more dynamic and interactive than merely consequential as historically described. Here we present a rare case of MS and ALS within the same patient and provide an overview of the complex interplay between neuroinflammation and neurodegeneration in these diseases.

## Case presentation

A 37 year old man presented with a left VIth cranial nerve palsy and nystagmus that improved on a brief course of steroids. A diagnosis of possible MS was entertained. Two years later, he experienced a subacute episode of bilateral leg weakness that was steroid responsive, prior to which he had a several month history of gradual decline in cognition, decreased visual acuity, widespread fasciculations with asymmetric upper limb weakness, and bilateral appendicular and truncal ataxia. The subsequent 12 months were notable for the evolution of debilitating lethargy and fatigue, and progressive dysarthria and dysphagia. He died of respiratory compromise at the age of 40.

### Pathology

**Figure 1:**
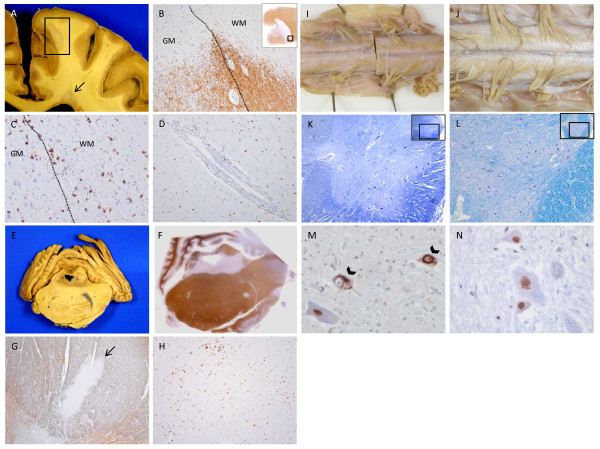
**Neuropathological features of concurrent MS and ALS.** Left hemispheric leukocortical (within box) and periventricular lesions (arrow) (A). PLP staining of leukocortical plaque in A (inlay) demonstrating demyelination spanning grey and white matter (B), PG-M1 staining showing macrophage infiltration and microglia activation (C), and perivascular cuffing with cells resembling lymphocytes (D) within same lesion. Gross (E) and PLP-stained section (F) of pons (with cerebellum) demonstrating periventricular and parenchymal demyelination. PLP-stained section of demyelinated lesion in dorsal column of cervical spinal cord (arrow) (G) with diffuse microglia activation extending beyond lesional borders (PG-M1 staining) (H). Gross view of thinned and discolored ventral roots of lower cervical cord from case (I) compared to control (J). LBCV section of lumbar cord demonstrating significant loss of anterior horn cells in the case (K) compared to control (L). TDP-43 staining demonstrating intracytoplasmic neuronal inclusions in anterior horn cells in lumbar spinal cord (arrowheads) in case (M) compared to nuclear neuronal staining of the same area in a control (N).

Formalin-fixed paraffin-embedded sections of pre-frontal and sub-frontal cortex, hippocampus, cerebellum, pons, low and high cervical and thoracic, lumbar and sacral spinal cord were stained with Luxol Fast Blue and Cresyl Violet (LBCV), Palmgren silver, Proteolipid protein (PLP), CD68 (PG-M1), CD3, and TDP-43 antibodies (Figure 1). Using the LCBV stain, anterior horn cells (AHC) were identified quantified and compared to counts from two age- and sex-matched controls using established methods.

Pathological confirmation of MS was supported by evidence of multiple discrete areas of inflammatory demyelination in prototypic locations, including cortex (subpial, leukocortical), periventricular region, corpus callosum (Figure 1A, B), pons (Figures 1E, F), cerebellar peduncle, and lateral and posterior columns of the spinal cord (Figure 1G). Microscopically, lesions were characterized by infiltration of macrophages in the epicenter of acute lesions and the border of chronic active lesions (Figure 1C); perivascular lymphocytic cuffing was noted in both (Figure 1D). Lesions of all stages of demyelinating activity were observed, with acute lesions being predominant. An extensive demyelinating lesion involving all layers of the hippocampus was the likely substrate for the patient's significant cognitive difficulty during life. Axonal density appeared to be mildly reduced in plaques when compared to surrounding normal appearing white matter. Meningeal inflammation with monocytic infiltrates was observed often without underlying subpial demyelination. No evidence of TDP-43 inclusions were noted in any of the MS lesions studied.

There were several classic features of ALS pathology in areas distinct from MS plaques. Ventral nerve roots were discoloured and selectively thinned (Figure 1I), and a marked reduction (approximately 75%) of anterior horn cells at multiple serial lumbosacral cord levels when compared to controls was observed (Figure 1K, L). Microglial activation was noted in i) layer V of the motor cortex (where Betz cells were sighted), and ii) at various levels of the spinal cord (Figure 1H). Corticospinal tract axonal loss appeared to be size selective, with large fibres particularly affected - a finding previously noted in ALS [1], and opposite to that found in MS (where small fibres are preferentially lost) [2]. Anterior horn cells in both cervical and lumbar cord demonstrated intracytoplasmic TDP-43 positive inclusions (Figure 1M), with no evidence of such inclusions in the cortical and deep gray matter regions examined. FUS staining was negative in all areas studied.

## Discussion

Post-mortem confirmation of concomitant ALS with MS has been verified only three times previously in the literature. Hader *et al*. [3] documented the case of a 56 year-old gentleman with a 27 year history of relapsing-remitting MS who, after 8 years of remission, developed bulbar signs with fasciculations, weakness and atrophy. Confavreaux *et al*. [4] described a similar story of a 25 year-old female who presented with MS and after a 10 year quiescent period again developed a similar range of ALS-like symptoms. Dynes *et al*. [5] described a case of a 62-year lady presenting with progressive quadriplegia and bulbar palsy who also experienced symptoms consistent with Lhermitte's phenomenon and paresthesia within an overlapping timeframe of just several months. Here we present yet another case of pathologically confirmed coincident MS and ALS and highlight the emerging overlap between these respectively viewed inflammatory and neurodegenerative diseases.

### i) Inflammation in MS and ALS

Whether inflammation is a cause or effect of the primary pathology, similar immunological profiles have been observed in both diseases. Both innate and adaptive arms of the immune response have been implicated in the pathogenesis of MS. Generalised activation of microglia, astrocytes and autoreactive T and B lymphocytes play a role in maintaining disease. There is evidence for involvement of specifically the Th17 population in perpetuating inflammation, in particular in association with production of IL-12 [6].

Pro-inflammatory cell populations are important in ALS too. RT-PCR in ALS patients of spinal cord white and gray matter has shown increased glial expression of toll-like receptor proteins [7]. Raised serum and CSF IL-12 and IL-17, levels in ALS patients, and high serum IL-23 levels point towards a similar cytokine and T-cell activation profile being present in both ALS and MS [8]. Increased levels of IL-13-producing T-cells correlate positively with ALS disease progression and severity. Clinically, it is intriguing that cannabinoid-receptor antagonists, through their anti-inflammatory effects, have been shown to be efficacious in the symptomatic management of not only MS, but also of ALS [9].

Inflammatory cell populations need not always be detrimental to neuronal survival, sometimes conferring a degree of neuroprotection. Microglia produce neurotrophic factors which directly assist with regeneration, such as neurotrophin 3 and brain-derived neurotrophic factor. There is recent evidence that activated T cell populations may also be crucial in neuroprotection, possibly in association with the secretion of IL-4 [10], associated with downstream production of insulin growth factor (IGF1). These protective inflammatory mechanisms may be important in both MS and ALS.

### ii) Degeneration in ALS and MS

Regardless of whether inflammation is a primary or secondary cause of pathology, the molecular basis of axonal loss in both diseases may converge on similar mechanisms. Microglia and astrocytes are instrumental in ALS models; selective knockout of microglia in SOD1 mutant mice has been shown to delay disease onset and progression. Glial cells are also hypothesised to amplify the degenerative process by activating NF-κβ by an inflammatory cytokine cascade, which incorporates the pro-apoptotic molecules FAS ligand and TDP-43 [11]. In the SOD1 models, nerve growth factor produced by activated astrocytes was associated with the death of motor neurons in the presence of NO and peroxide production [12]. Microglial cells may form part of a degradation pathway mediated by excitotoxicity as alterations in metabotropic glutamate receptor expression have been seen in human spinal cord [13].

Similar to ALS, MS pathology demonstrates activated microglia and astrocytes known to produce NO and reactive oxygen species. In MS, the presence of these reactive species may lead to degeneration of both the neuron and myelin sheath. Secretion of IL-23 and osteopontin also leads to downstream production of TNF-α, promoting myelin degradation [6]. The activation of NF-κβ via an inflammatory cytokine cascade initiated and propagated by microglia and astrocytes has also been shown to occur in MS with the downstream consequence of neuronal/axonal degeneration. Glutamate excitotoxicity is increasingly recognized as an important feature of both myelin and axonal destruction in MS, a feature which has prompted the use of anti-glutaminergic agents often used in ALS, such as riluzole, in experimental animal models of MS [14].

## Conclusions

We present an unusual case of a pathologically confirmed coincident MS and ALS. Axonal loss, the substrate for irreversible neurological disability, is a shared pathologic feature in both conditions with intriguing similarities emerging in the molecular and cellular pathways leading to axonal demise. Given the underwhelming impact of current therapies to prevent disability in MS and ALS, efforts directed at understanding the complex interplay between inflammation and degeneration in both MS and ALS will be essential to develop therapies designed to halt the devastating consequences of these diseases.

## Consent

Consent for use of archival post-mortem tissue prior to 2006 is given for research activity on tissue at the Thomas Willis Oxford Brain Collection where no explicit objection for research use is given by the patient or next of kin (as in the case of this patient report) according to regulations outlined in the UK Human Tissue Act.

## List of abbrevations

MS: multiple sclerosis; ALS: amyotrophic lateral sclerosis; TDP-43: TAR DNA-binding protein 43; IL: interleukin; IGF: insulin growth factor; SOD: superoxide dismutase; NO: nitric oxide; TNF: tumour necrosis factor.

## Competing interests

The authors declare that they have no competing interests.

## Authors' contributions

GD and MME conceived the design of the study. GL and GD acquired, analysed, and interpreted the data and drafted the manuscript. GD, OA, and MME revised the manuscript critically for important intellectual content and gave final approval of the version to be published. All authors read and approved the final manuscript.

## References

[B1] Sobue G Hashizume Y Mitsuma T Takahashi A Size-dependent myelinated fiber loss in the corticospinal tract in Shy-Drager syndrome and amyotrophic lateral sclerosis Neurology 1987 37 3 529 32 3822153 10.1212/wnl.37.3.529

[B2] DeLuca GC Ebers GC Esiri MM Axonal loss in multiple sclerosis: a pathological survey of the corticospinal and sensory tracts Brain 2004 127 1009 18 10.1093/brain/awh118 15047586

[B3] Hader WJ Rpzdilsky B Nair CP The concurrence of multiple sclerosis and amyotrophic lateral sclerosis Can J Neurol Sci 1986 13 66 9 3955455 10.1017/s0317167100035824

[B4] Confavreux C Moreau T Jouvet A Association of amyotrophic lateral sclerosis and multiple sclerosis Rev Neurol 1986 149 351 3 8272733

[B5] Dynes GJ Schwimer CJ Staugaitis SM Doyle JJ Hays AP Mitsumoto H Amyotrophic lateral sclerosis with multiple sclerosis: a clinical and pathological report Amyotroph Lateral Scler Other Motor Neuron Disord 2000 1 349 53 10.1080/146608200750139837 11464854

[B6] Korn T Bettelli E Oukka M Kuchroo VK IL-17 and Th17 Cells Annu Rev Immunol 2009 27 485 517 10.1146/annurev.immunol.021908.132710 19132915

[B7] Casula M Iyer AM Spliet WG Anink JJ Steentjes K Sta M Troost D Aronica E Toll-like receptor signaling in amyotrophic lateral sclerosis spinal cord tissue Neuroscience 2011 179 233 43 21303685 10.1016/j.neuroscience.2011.02.001

[B8] Rentzos M Rombos A Nikolaou C Zoga M Zouvelou V Dimitrakopoulos A Alexakis T Tsoutsou A Samakovli A Michalopoulou M Evdokimidis I Interleukin-15 and interleukin-12 are elevated in serum and cerebrospinal fluid of patients with amyotrophic lateral sclerosis Eur Neurol 2010 63 285 90 10.1159/000287582 20407265

[B9] Rossi S Bernardi G Centonze D The endocannabinoid system in the inflammatory and neurodegenerative processes of multiple sclerosis and of amyotrophic lateral sclerosis Exp Neurol 2010 224 92 102 10.1016/j.expneurol.2010.03.030 20353778

[B10] Beers DR Henkel JS Zhao W Wang J Huang A Wen S Liao B Appel SH Endogenous regulatory T lymphocytes ameliorate amyotrophic lateral sclerosis in mice and correlate with disease progression in patients with amyotrophic lateral sclerosis Brain 2011 134 Pt 5 1293 314 21596768 10.1093/brain/awr074 PMC3097891

[B11] Raoul C Buhler E Sadeghi C Jacquier A Aebischer P Pettmann B Henderson CE Haase G Chronic activation in presymptomatic amyotrophic lateral sclerosis (ALS) mice of a feedback loop involving Fas, Daxx, and FasL Proc Natl Acad Sci USA 2006 103 6007 12 10.1073/pnas.0508774103 16581901 PMC1458688

[B12] Pehar M Cassina P Vargas MR Castellanos R Viera L Beckman JS Estévez AG Barbeito L Astrocytic production of nerve growth factor in motor neuron apoptosis: implications for amyotrophic lateral sclerosis J Neurochem 2004 89 464 73 10.1111/j.1471-4159.2004.02357.x 15056289

[B13] Aronica E Catania MV Geurts J Yankaya B Troost D Immunohistochemical localization of group I and II metabotropic glutamate receptors in control and amyotrophic lateral sclerosis human spinal cord: upregulation in reactive astrocytes Neuroscience 2001 105 509 20 10.1016/S0306-4522(01)00181-6 11672616

[B14] Gilgun-Sherki Y Panet H Melamed E Offen D Riluzole suppresses experimental autoimmune encephalomyelitis: implications for the treatment of multiple sclerosis Brain Res 2003 989 196 204 10.1016/S0006-8993(03)03343-2 14556941

